# Red and White Meat Intake in Relation to Mental Disorders in Iranian Adults

**DOI:** 10.3389/fnut.2021.710555

**Published:** 2021-07-27

**Authors:** Shiva Kazemi, Ammar Hassanzadeh Keshteli, Parvane Saneei, Hamid Afshar, Ahmad Esmaillzadeh, Peyman Adibi

**Affiliations:** ^1^Department of Community Nutrition, School of Nutritional Sciences and Dietetics, Tehran University of Medical Sciences, Tehran, Iran; ^2^Food Security Research Center, Isfahan University of Medical Sciences, Isfahan, Iran; ^3^Department of Medicine, University of Alberta, Edmonton, AB, Canada; ^4^Isfahan Gastroenterology and Hepatology Research Center, Isfahan University of Medical Sciences, Isfahan, Iran; ^5^Department of Community Nutrition, School of Nutrition and Food Scienc, Isfahan University of Medical Sciences, Isfahan, Iran; ^6^Department of Psychiatry, Psychosomatic Research Center, Isfahan University of Medical Sciences, Isfahan, Iran; ^7^Obesity and Eating Habits Research Center, Endocrinology and Metabolism Molecular-Cellular Sciences Institute, Tehran University of Medical Sciences, Tehran, Iran

**Keywords:** meat intake, mental disorder, depression, anxiety, psychological distress, diet

## Abstract

**Background:** The association between meat consumption and mental disorders is less investigated in Iranian population. We examined the association between meat consumption and prevalence of symptoms of depression, anxiety, and psychological distress in Iranian adults.

**Methods:** This cross-sectional study included 3,362 participants aged 18–55 years old. A dish-based 106-item semiquantitative food frequency questionnaire (FFQ) was used to assess usual dietary intake of study population. Hospital Anxiety and Depression Scale (HADS) and General Health Questionnaire (GHQ), all validated in Iranian population, were applied to collect data on symptoms of anxiety, depression, and psychological distress, respectively.

**Results:** The prevalence of symptoms of depression, anxiety, and psychological distress in the study population was 28.6, 13.6, and 22.6%, respectively. After considering potential confounders, individuals in the top quartile of red meat intake had 43% increased risk of depression symptoms [odds ratio (OR) = 1.43; 95% CI: 1.09–1.89] compared to those in the first quartile. No significant relation was observed between red meat intake and anxiety or psychological distress symptoms. White meat consumption was not associated with mental disorders. Stratified analysis by sex showed that male participants in the highest quartile of red meat intake had 92% greater risk of depression symptoms (95% CI: 1.17–3.15) than those individuals in the lowest category. Red and white meat intake was not associated with mental disorders in women. In overweight or obese individuals, despite lack of any association between red meat intake and mental disorders, high intake of white meat was associated with a lower odds of psychological distress symptoms (OR = 0.64; 95% CI: 0.42–0.99) and a lower risk of depression symptoms (OR = 0.68; 95% CI: 0.45–1.00). In normal-weight participants, those in the highest quartile of red meat intake had greater odds for depression symptoms than those in the lowest quartile (OR = 1.66; 95% CI: 1.14–2.42).

**Conclusions:** We found that red meat consumption was associated with increased risk of depression symptoms, especially in men, and normal-weight participants. In overweight or obese participants, white meat intake was inversely associated with psychological distress symptoms.

## Introduction

Mental disorders are a growing public health concern (1). Anxiety and depression are two most common mental disorders (2). In 2015, World Health Organization (WHO) reported that 44.3 million people suffer from depression, and 37.3 million people suffer from anxiety worldwide (3). The prevalence of anxiety and depression symptoms among Iranian general population is estimated to be 42 and 37.2%, respectively (4–6). Previous studies on different populations demonstrated that anxiety and depression were associated with an increased risk of several chronic diseases, including hemorrhagic stroke, myocardial infarction, diabetes, cancer, and irritable bowel syndrome (7–10). The economic burden of depression in the US was estimated to be 83.1 billion dollars in 2000 and 118 billion dollars in 2004 in Europe (11, 12).

Several modifiable and non-modifiable risk factors were associated with anxiety and depression prevalence rates (4, 13). For example, unhealthy dietary intake could significantly increase the risk of mental disorders (14–16). Red or processed meat intake has rarely been investigated in relation to psychological disorders (17–20). Given that red or processed meat consumption is associated with elevated levels of pro-inflammatory cytokines, they might also involve in the development of mental disorders (8).

A recently published meta-analysis, which included three cross-sectional, three cohort, and two case–control studies, examined the relation between meat consumption and depression (17). Most included cross-sectional and case–control studies in that meta-analysis revealed no significant association between meat consumption and depression (21–26), whereas, included cohort studies (26–28) reported that meat consumption was associated with a 13% greater risk of depression (17). It is noteworthy that some cross-sectional studies reported increased risk of depression among individuals with a rare meat consumption as less than once a week compared to moderate levels of intake (21, 23). Higher intake of meat, compared to moderate consumption, was also associated with a high prevalence of depression (23). There is no investigation in this regard in Iranian population. Given the high prevalence of depressive symptoms among Iranians and the different dietary pattern of this population compared to others, the aim of this investigation was to evaluate the association between red and white meat intake and symptoms of mental disorders in a large group of Iranian adults.

## Materials and Methods

### Study Design

The data of this cross-sectional study was derived from the Study on the Epidemiology of Psychological-Alimentary Health and Nutrition (SEPAHAN), a cross-sectional study started in 2010, with two main goals of assessing the link of functional gastrointestinal disorders (FGIDs) and their symptoms with lifestyle and nutritional aspects, and also psychological features. The study was carried out among the Iranian adults with the age of 18–55 years, who worked in 50 health centers affiliated to Isfahan University of Medical Sciences (IUMS). In the first step, questionnaires on sociodemographic factors, dietary habits, and dietary intake were sent to 10,087 individuals, a group of 8,691 participants returned the questionnaires (response rate: 86.16%). In the second step, which was done 1 month later, a validated questionnaire on psychological distress and mental disorders was distributed among the study population (response rate: 64.64%). When the demographic data between individuals who returned the completed questionnaires in the first step was compared to those who returned the completed questionnaires in both phases, no significant difference was found. After integrating data from the two steps, complete information was available for 3,846 participants. Some data were not usable either due to incomplete questionnaires in step 1 or 2 (*n* = 998, 9.9%), or lack of identification code in one of these steps (*n* = 884, 9.0%), or incomplete questionnaires on the exposure, outcome, or covariates (*n* = 789, 7.6%). Moreover, participants who reported their calorie intake outside the range of 800–4,200 kcal/day were excluded (*n* = 484, 4.8%) (29) because energy intake outside this range is unlikely to be true for relatively inactive women and active men (29). Finally, data of 3,362 adults were used for the present analysis. Written informed consent was obtained from each participant. The study protocol was approved by the bioethics committee of Food Security Research Center, Isfahan University of Medical Sciences, Iran (no. 299021 and ethical code IR.MUI.RESEARCH.REC.1399.221).

### Assessment of Meat Intake

We used a validated dish-based 106-item semiquantitative food frequency questionnaire (FFQ), designed for Iranian adults, to compute red and white meat intake (30). This FFQ included five groups of dishes and foods; two of these categories—mixed dishes (cooked or canned, 29 items) and other food items (fast foods and other miscellaneous foods, 36 items)—were used to calculate meat intake of the participants. In brief, participants were asked to report their frequency of dietary intake of foods and mixed dishes over the last year, with nine various responses from “never or less than once a month” to “12 or more times per day.” Then, the daily grams of each food item were calculated using the household measures. An Iranian-version of Nutritionist IV software (First Databank, San Bruno, CA, USA) was used to achieve the daily average nutrient intake for each participant. The applied FFQ could reasonably classify usual intake of foods and food groups in Iranian adults, based on our previous investigations (31, 32). In this study, red meat consumption was calculated by summing up the intake of red meat (beef, veal, mutton, and lamb), processed meat (sausages, hamburgers, and hot dogs), and visceral meats (lamb's liver, kidney, and heart). White meat consumption included all kinds of fish, chicken, and poultry.

### Assessment of Outcomes

To assess the anxiety and depression symptoms, we used Hospital Anxiety and Depression Scale (HADS), which was validated in Iranian population (33). HADS is a short and efficient questionnaire to assess psychological disorders symptoms and to determine the scale of the symptoms of anxiety disorders and depression. It includes 14 items and composes of two sections: anxiety and depression. Each item includes a Likert-type scale (0–1–2–3), in the present study in both sections, anxiety and depression symptoms, the points of 0–7 were considered as “normal” and the points of 8 or more were interpreted as having symptoms of psychological disorders. The Cronbach's alpha coefficient was 0.78 for anxiety subscale and 0.86 for depression subscale, based on the validation study of HADS among Iranian adults (33). To define psychological distress symptoms, we used an Iranian-validated version of General Health Questionnaire (GHQ), a short and simple to use questionnaire that has 12 items with a Likert-type scale (less than usual, no more than usual, rather more than usual, or much more than usual). Responders were asked to report whether they recently have a specific symptom of psychological distress. In the current study, the bimodal scoring method with the score of 0–0–1–1 was applied and provided scores ranging from 0 to 12. Scoring of 4 or more was defined as having symptoms of high levels of psychological distress. Lower scores represented low levels of psychological distress in individuals. The Cronbach's alpha coefficient for psychological distress among Iranians was 0.87 (34).

### Assessment of Confounders

To obtain data on age, sex, education, marital status, smoking status, family size, socioeconomic status (SES), diabetes, antidepressant medication, and dietary supplement use, a self-administered questionnaire was applied. Anthropometric information (including height and weight) was collected by using a validated self-reported questionnaire (35). Body mass index (BMI) was calculated as weight in kilograms divided by the square of the height in meters. Participants were divided into two groups according to their BMI: normal weight (<25 kg/m^2^) and overweight or obese (≥25 kg/m^2^). General Practice Physical Activity Questionnaire (GPPAQ), a valid simple four-level physical activity index, was used to estimate the activity levels of participants (36).

### Statistical Methods

First, we obtained energy-adjusted amounts of red and white meat intake through residual method. Then, participants were classified based on energy-adjusted quartiles of red and white meat intake. As it is common in nutritional epidemiology (29), we used quartiles to compare individuals with the highest intake vs. individuals with the lowest intake in relation to the outcomes of the interests. One-way ANOVA was used to compare of continuous variables across different categories of red and white meat intake. Chi-square test was applied to examine the distribution of categorical variables across different categories of red and white meat intake. Analysis of covariance (ANCOVA) with the Bonferroni correction was used to report the mean intake of nutrients and food groups after adjustment of age, sex, and energy intake. The logistic regression was used to assess the relation between red and white meat intake and psychological disorders symptoms. The relations were first examined in crude model. Then, adjustment was done for the confounder variables, including age (years), sex (male/female), energy intake (kcal/day), physical activity (≥1/ <1 h/week), smoking (current smokers/ex-smokers/non-smokers), marital status (single/married), SES [consist of educational level (>diploma/ ≤ diploma), family size (>4/ ≤ 4 members), and house ownership (yes/no)], self-reported diabetes (yes/no), use of antidepressants medications (yes/no), and dietary supplements (yes/no) in Model 1. Dietary intake (including intake of fat, dairy, nuts, soy and legumes, grains, fruit, vegetables, and *n-*3 fatty acids) and BMI were also considered in Model 2. To determine the trend of odds ratios (ORs) across different levels of red and white meat intake, we considered the quartiles of meat as an ordinal variable. The participants in the first category of meat intake were considered as the reference category in all models. The analyses were also conducted separately by sex and BMI status. Statistical Package for Social Sciences (SPSS Inc., version 18.0, Chicago, IL, USA) was used for all analyses and *p*-values <0.05 were considered statistically significant.

## Results

Mean age and weight of study participants were 36.29 ± 7.87 (SD) years and 68.65 ± 13.18 kg, respectively. The prevalence of symptoms of depression, anxiety, and psychological distress in the study population was 28.6, 13.6, and 22.6%, respectively. General characteristics of study participants and the prevalence of symptoms of depression, anxiety, and high psychological distress across quartiles of energy-adjusted red and white meat intake are provided in Table 1. Participants in highest quartile of red meat intake had higher weight (69.75 ± 14.25 vs. 68.33 ± 12.61, *p*-value = 0.02), physical activity (16.00 vs. 11.20%, *p*-value = 0.03), and were less likely to be women (52 vs. 58.5%, *p*-value <0.001), educated (54.30 vs. 66.70%, *p*-value <0.001), and more likely to be homeowners (59 vs. 56.70%, *p*-value = 0.02), compared with those in the lowest quartile. Individuals in the top category of white meat intake had higher weight (69.93 ± 13.13 vs. 68.84 ± 13.04, *p*-value <0.001) and physical activity (33 vs. 24.40%, *p*-value <0.001) and were less likely to be women (50.80 vs. 54.60%, *p*-value <0.001), married (78.80 vs. 80.20%, *p*-value = 0.01) compared with those in the bottom category. There were no significant differences in other demographic characteristics of participants across quartiles of red and white meat intake. Higher prevalence of depression symptoms (32.6 vs. 25.2%, *p*-value = 0.01) was observed among individuals in the top quartile of red meat intake compared with those in the bottom quartile. There was no significant difference across quartiles of meat intake with regard to anxiety and psychological distress symptoms. No significant differences were found in the prevalence of symptoms of mental disorders between different levels of white meat intake.

**Table 1:** General characteristics of study participants and prevalence of symptoms of depression, anxiety, and high psychological distress across quartiles of energy-adjusted red and white meat intake^a^.

	**Quartiles of energy-adjusted red meat intake**					**Quartiles of energy-adjusted white meat intake**				
	**Q1** **(*n* = 840)**	**Q2** **(*n* = 841)**	**Q3** **(*n* = 841)**	**Q4** **(*n* = 840)**	***p*-value^**b**^**	**Q1** **(*n* = 840)**	**Q**2 **(*n* = 841)**	**Q3** **(*n* = 841)**	**Q4** **(*n* = 840)**	***p*-value^**b**^**
Age (years)	36.29 ± 7.74	36.07 ± 7.77	35.94 ± 7.73	36.87 ± 8.23	0.11	36.59 ± 7.84	36.14 ± 7.66	35.74 ± 7.51	36.72 ± 8.44	0.07
Weight (kg)	68.33 ± 12.61	67.80 ± 12.51	68.71 ± 13.23	69.75 ± 14.25	0.02	68.84 ± 13.04	67.70 ± 13.09	68.15 ± 13.39	69.93 ± 13.13	<0.001
BMI (kg/m^2^)	24.85 ± 3.69	24.82 ± 3.80	24.92 ± 3.94	25.03 ± 3.86	0.70	24.85 ± 3.82	24.86 ± 3.75	24.92 ± 3.84	24.99 ± 3.88	0.88
Female, *n* (%)	491 (58.5)	522 (62.1)	509 (60.5)	437 (52.0)	<0.001	459 (54.6)	532 (63.3)	541 (64.3)	427 (50.8)	<0.001
Married, *n* (%)	671 (79.9)	683 (81.2)	696 (82.8)	696 (82.9)	0.60	674 (80.2)	713 (84.8)	697 (82.9)	662 (78.8)	0.01
Education, *n* (%) (>diploma)	560 (66.7)	558 (66.3)	507 (60.3)	456 (54.3)	<0.001	684 (57.9)	514 (61.1)	542 (64.4)	509 (60.6)	0.11
Family size, *n* (%) (>4)	108 (12.9)	106 (12.6)	90 (10.7)	123 (14.6)	0.12	131 (15.6)	93 (11.1)	95 (11.3)	108 (12.9)	0.02
House possession, *n* (%)	476 (56.7)	(57.0)	508 (60.4)	496 (59.0)	0.02	475 (56.5)	481 (57.2)	505 (60.0)	498 (59.3)	0.23
Diabetes, *n* (%)	15 (1.8)	8 (1.0)	18 (2.1)	19 (2.3)	0.17	14 (1.7)	15 (1.8)	9 (1.1)	22 (2.6)	0.12
Anti-psychotic medications^c^, *n* (%)	42 (5.0)	40 (4.8)	55 (6.5)	50 (6.0)	0.35	56 (6.7)	48 (5.7)	43 (5.1)	40 (4.8)	0.34
Dietary supplement use^d^, *n* (%)	260 (31.0)	262 (31.2)	244 (29.0)	243 (28.9)	0.63	232 (27.6)	279 (33.2)	276 (32.8)	222 (26.4)	<0.001
Smokers, *n* (%)	108 (12.9)	112 (13.3)	107 (12.7)	137 (16.3)	0.11	109 (13.0)	98 (11.7)	121 (14.4)	136 (16.2)	0.05
Physically active, *n* (%) (≥1 h/week)	94 (11.2)	104 (12.4)	111 (13.2)	134 (16.0)	0.03	108 (12.9)	85 (10.1)	104 (12.4)	146 (17.4)	<0.001
Obese^e^, *n* (%)	535 (63.7)	372 (44.2)	383 (45.5)	384 (45.7)	0.80	384 (45.7)	374 (44.5)	378 (44.8)	371 (44.2)	0.93
Prevalence of depression symptoms, *n* (%)	212 (25.2)	237 (28.2)	239 (28.4)	274 (32.6)	0.01	244 (29.0)	231 (27.5)	257 (30.6)	229 (27.3)	0.42
Prevalence of anxiety symptoms, *n* (%)	106 (12.6)	124 (14.7)	100 (11.9)	127 (15.1)	0.16	110 (13.1)	120 (14.3)	123 (14.6)	104 (12.4)	0.54
Prevalence of high psychological distress symptoms, *n* (%)	191 (22.7)	192 (22.8)	187 (22.2)	190 (22.6)	0.99	197 (23.5)	193 (22.9)	200 (23.8)	170 (20.2)	0.29

a *All values are means ± SD, unless indicated*.

b *Obtained from ANOVA for continuous variables and chi-square test for categorical variables*.

c *Anti-psychotic medications include nortriptyline, amitriptyline or imipramine, fluoxetine, citalopram, fluvoxamine, and sertraline*.

d *Dietary supplements include iron, calcium, vitamins, and other dietary supplements*.

e *BMI ≥ 25*.

Dietary intake of selected nutrients and food groups of study participants across quartiles of energy-adjusted red and white meat intake is shown in Table 2. In the highest quartile of red meat intake, compared with the lowest quartile, we observed lower intake of energy, carbohydrates, vitamin B_1_, iron, whole grains, refined grains, and fruits, but higher intake of proteins, fats, omega-3 fatty acids, vitamin B_6_, vitamin E, vegetables, nuts, soy, and legumes. Individuals with the highest intake of white meat had higher consumption of proteins, fats, omega-3 fatty acids, vitamin B_6_, vitamin C, vitamin E, vegetables, nuts, soy and legumes and significantly lower intake of energy, carbohydrates, dietary fiber, vitamin B_1_, iron, whole grains, and refined grains compared with those in the lowest quartile.

**Table 2:** Dietary intake of selected nutrients and food groups of study participants across quartiles of energy-adjusted red and white meat intake^a^.

	**Quartiles of red meat intake**					**Quartiles of white meat intake**				
	**Q1** **(*n* = 840)**	**Q2** **(*n* = 841)**	**Q3** **(*n* = 841)**	**Q4** **(*n* = 840)**	***p*-value^**b**^**	**Q1** **(*n* = 840)**	**Q2** **(*n* = 841)**	**Q3** **(*n* = 841)**	**Q4** **(*n* = 840)**	***p*-value^**b**^**
Energy (kcal/day)	2641.32 ± 28.5	2134.87 ± 28.8	2189.80 ± 28.8	2561.03 ± 29.0	<0.001	2730.88 ± 28.3	2172.32 ± 28.1	2102.78 ± 28.4	2531.62 ± 28.8	<0.001
**Nutrients**										
Proteins (% of energy)	13.80 ± 0.08	14.28 ± 0.08	15.17 ± 0.08	16.11 ± 0.08	<0.001	13.36 ± 0.07	13.98 ± 0.07	15.05 ± 0.07	17.01 ± 0.07	<0.001
Fats (% of energy)	32.75 ± 0.20	35.87 ± 0.20	39.03 ± 0.20	42.50 ± 0.20	<0.001	34.26 ± 0.22	36.65 ± 0.22	38.34 ± 0.22	40.86 ± 0.22	<0.001
Carbohydrates (% of energy)	54.83 ± 0.23	51.35 ± 23	47.29 ± 0.23	42.94 ± 234	<0.001	53.91 ± 0.25	50.92 ± 0.25	48.10 ± 0.25	43.48 ± 0.25	<0.001
Dietary fiber (g/day)	22.72 ± 0.21	22.72 ± 0.21	22.63 ± 0.21	22.40 ± 0.21	0.65	23.74 ± 0.21	22.83 ± 0.21	22.41 ± 0.21	21.46 ± 0.21	<0.001
Omega-3 fatty acids (g/day)	1.69 ± 0.03	1.72 ± 0.03	1.76 ± 0.03	1.82 ± 0.03	<0.001	1.53 ± 0.03	1.67 ± 0.03	1.78 ± 0.03	2.00 ± 0.03	<0.001
Vitamin B1 (mg/day)	2.23 ± 0.02	1.90 ± 0.02	1.76 ± 0.02	1.50 ± 0.02	<0.001	2.14 ± 0.02	1.87 ± 0.02	1.79 ± 0.02	1.59 ± 0.02	<0.001
Vitamin B6 (mg/day)	1.68 ± 0.01	1.90 ± 0.01	2.05 ± 0.01	2.31 ± 0.01	<0.001	1.81 ± 0.01	1.94 ± 0.01	2.01 ± 0.01	2.18 ± 0.01	<0.001
Iron (mg/day)	18.57 ± 0.12	17.73 ± 0.12	17.38 ± 0.12	16.77 ± 0.12	<0.001	18.80 ± 0.12	17.67 ± 0.12	17.39 ± 0.12	16.57 ± 0.12	<0.001
Vitamin C (mg/day)	100.18 ± 1. 89	103.83 ± 1.91	103.30 ± 1.90	99.62 ± 1.91	0.31	97.30 ± 1.92	101.07 ± 1.88	101.31 ± 1.91	107.43 ± 1.91	<0.001
Vitamin E (mg/day)	16.31 ± 0.17	20.35 ± 0.17	22.74 ± 0.17	26.55 ± 0.17	<0.001	19.10 ± 0.21	21.17 ± 0.21	22.04 ± 0.21	23.55 ± 0.21	<0.001
**Food groups (g/day**)										
Whole grains	54.48 ± 2.74	47.12 ± 2.77	41.02 ± 2.75	27.27 ± 2.77	<0.001	47.54 ± 2.81	47.11 ± 2.75	40.99 ± 2.79	34.33 ± 2.80	<0.001
Refined grains	446.76 ± 5.85	402.46 ± 5.90	380.41 ± 5.87	340.23 ± 5.91	<0.001	451.64 ± 5.93	401.54 ± 5.80	381.24 ± 5.89	335.19 ± 5.91	<0.001
Fruit	337.08 ± 8.28	339.67 ± 8.36	311.22 ± 8.32	282.47 ± 8.37	<0.001	318.52 ± 8.48	318.3 ± 8.29	315.80 ± 8.43	318.58 ± 8.46	0.96
Vegetables	215.96 ± 4.24	228.75 ± 4.28	248.04 ± 4.26	265.14 ± 4.28	<0.001	224.04 ± 4.36	238.85 ± 4.26	242.00 ± 4.33	252.67 ± 4.35	<0.001
Nuts, soy, and legumes	44.35 ± 1.28	56.08 ± 1.30	60.03 ± 1.29	68.90 ± 1.30	<0.001	51.91 ± 1.34	57.54 ± 1.31	57.76 ± 1.33	61.91 ± 1.34	<0.001
High fat dairy	14.39 ± 0.63	14.42 ± 0.64	14.53 ± 0.63	15.52 ± 0.64	0.54	14.43 ± 0.64	15.11 ± 0.63	15.18 ± 0.64	14.10 ± 0.64	0.59

a *All values are means ± SE, energy intake is adjusted for age and sex; all other values are adjusted for age, sex, and energy intake*.

b *Obtained from ANCOVA*.

Multivariable-adjusted ORs and (95% CI) for depression, anxiety, and psychological distress symptoms across quartiles of energy-adjusted red and white meat intake in the whole study population are presented in Table 3. The risk of depression symptoms among individuals in the top quartile of red meat intake was 43% greater than those in the first quartile (OR = 1.43; 95% CI: 1.16–1.78). The association remained significant even after controlling for all potential confounders including BMI (OR = 1.43; 95% CI: 1.09–1.89). No significant relation was observed between red meat intake and anxiety or psychological distress symptoms. Similarly, white meat intake was not associated with depression, anxiety, and psychological distress symptoms either in crude or adjusted models.

**Table 3:** Multivariable-adjusted odds ratios and 95% confidence intervals for depression, anxiety, and psychological distress symptoms across quartiles of energy-adjusted red and white meat intake in whole population^a^.

	**Quartiles of red meat intake**					**Quartiles of white meat intake**				
	**Q1** **(*n* = 840)**	**Q2** **(*n* = 841)**	**Q3** **(*n* = 841)**	**Q4** **(*n* = 840)**	***p*_**trend**_**	**Q1** **(*n* = 840)**	**Q2** **(*n* = 841)**	**Q3** **(*n* = 841)**	**Q4** **(*n* = 840)**	***p*_**trend**_**
**Depression symptoms**										
Crude	1.00	1.17 (0.94–1.45)	1.18 (0.95–1.46)	1.43 (1.16–1.78)	<0.001	1.00	0.93 (0.75–1.15)	1.08 (0.87–1.33)	0.92 (0.74–1.14)	0.77
Model 1	1.00	1.19 (0.92–1.52)	1.18 (0.92–1.52)	1.47 (1.16–1.88)	<0.001	1.00	0.96 (0.75–1.23)	1.18 (0.92–1.51)	0.93 (0.72–1.19)	0.96
Model 2	1.00	1.15 (0.88–1.50)	1.13 (0.87–1.48)	1.43 (1.09–1.89)	0.02	1.00	0.95 (0.73–1.23)	1.16 (0.89–1.51)	0.89 (0.68–1.17)	0.79
**Anxiety symptoms**										
Crude	1.00	1.19 (0.90–1.58)	0.93 (0.69–1.25)	1.23 (0.93–1.63)	0.38	1.00	1.10 (0.83–1.46)	1.13 (0.85–1.50)	0.94 (0.70–1.25)	0.73
Model 1	1.00	1.07 (0.78–1.48)	0.80 (0.57–1.12)	1.13 (0.82–1.55)	0.87	1.00	1.06 (0.77–1.47)	1.18 (0.85–1.64)	0.91 (0.65–1.27)	0.76
Model 2	1.00	1.10 (0.78–1.54)	0.89 (0.62–1.28)	1.27 (0.89–1.83)	0.39	1.00	1.11 (0.79–1.55)	1.23 (0.87–1.74)	0.96 (0.67–1.40)	0.97
**Psychological distress symptoms**										
Crude	1.00	1.01 (0.80–1.26)	0.97 (0.77–1.22)	0.99 (0.79–1.25)	0.88	1.00	0.97 (0.78–1.22)	1.02 (0.81–1.28)	0.83 (0.66–1.04)	0.17
Model 1	1.00	1.02 (0.79–1.33)	1.00 (0.77–1.29)	1.03 (0.79–1.33)	0.91	1.00	0.96 (0.74–1.25)	1.12 (0.86–1.46)	0.88 (0.68–1.15)	0.60
Model 2	1.00	1.01 (0.76–1.32)	1.02 (0.77–1.34)	1.03 (0.77–1.38)	0.84	1.00	0.93 (0.71–1.22)	1.14 (0.87–1.51)	0.87 (0.65–1.17)	0.71

a *Model 1: Adjusted for age, sex, energy intake, physical activity, smoking, marital status, education, socioeconomic status (SES), diabetes, intake of Anti-psychotic medications, and dietary supplements. Model 2: Additional controlling for dietary intake of high fat dairy, nuts, soy and legumes, grains, fruit and vegetables, n−3 fatty acids, and BMI*.

Multivariable-adjusted ORs and 95% CI for depression, anxiety, and psychological distress symptoms across quartiles of energy-adjusted red and white meat intake in men and women are provided in Supplemental Tables 1, 2, respectively. Male participants in the highest quartile of red meat intake had higher odds of depression symptoms (OR = 1.75; 95% CI: 1.23–2.51) compared with those in the lowest quartile. After controlling for potential confounders, this association was strengthened (OR = 1.92; 95% CI: 1.17–3.15). Either in crude or in the adjusted models, no significant associations were observed between different levels of red meat intake and odds of anxiety and psychological distress symptoms among male participants. In case of white meat intake, we found that men in the third quartile had higher risk of depression symptoms (OR = 1.64; 95% CI: 1.02–2.63) and psychological distress symptoms (OR = 2.02; 95% CI: 1.20–3.39) compared to those in the first quartile.

Women in the highest quartile of red meat intake had greater odds of depression symptoms (OR = 1.36; 95% CI: 1.04–1.79) than those in the lowest quartile. However, after controlling for dietary intake, omega-3 fatty acids, and BMI, no statistically significant association was seen. No significant association was observed between different levels of red meat intake and odds of anxiety and psychological distress symptoms in women. Moreover, no significant associations were found between different amount of white meat intake and mental disorders symptoms after considering all potential confounders.

Multivariable-adjusted ORs and 95% CI for psychological disorders across quartiles of energy-adjusted red and white meat intake in overweight or obese participants (BMI ≥ 25 kg/m^2^) and normal-weight participants (BMI <25 kg/m^2^) are presented in Supplemental Tables 3, 4, respectively. After adjustment for confounding variables, no association was found between red meat intake and symptoms of mental disorders in overweight or obese individuals. However, higher intake of white meat was significantly associated with lower odds of psychological distress symptoms (OR = 0.64; 95% CI: 0.42–0.99) and marginally associated with lower risk of depression symptoms (OR = 0.68; 95% CI: 0.45–1.00) compared to lower intake.

In normal-weight participants, highest quartiles of red meat intake associated with higher odds of depression symptoms in both crude (OR = 1.44; 95% CI: 1.08–1.92) and fully adjusted model (OR = 1.66; 95% CI: 1.14–2.42). No significant relation was observed between white meat intake and symptoms of mental disorders in normal-weight participants.

## Discussion

This cross-sectional study showed a significant positive association between red meat intake and the risk of depression symptoms in Iranian adults. This association was seen among male participants, even after adjustment for all potential confounders. But there was no relation in females, after considering confounding factors. In addition, in normal-weight participants, a significant positive association was observed between red meat intake and the odds of depression symptoms. However, analysis in overweight or obese participants showed that white meat intake had an inverse association with odds of psychological distress symptoms and marginally inverse association with depression symptoms. To our knowledge, this study was one of the first investigations in the Middle East, which evaluated the relation between meat intake and mental disorders.

Considering the high prevalence of mental disorders, especially depression all over the world, even a minimal advantage to reduce the prevalence of depression or anxiety would be substantial to the entire population. Our results showed that red and white meat intake might be associated with some mental disorders symptoms.

Previous studies have shown inconsistent results on relation between depression and meat consumption. Two prospective cohort studies indicated a significant association between meat or processed meat consumption (but not fish intake) and depression (26, 27). Whitehall II, another cohort study conducted on 3,486 participants, showed that high adherence to “processed food dietary pattern” including processed meat was associated with an increased odds of depression (37). Similarly, a pilot randomized controlled trials have suggested that restriction of meat, fish, and poultry consumption could improve mental state (38). Zhou et al. (23) in a cross-sectional study indicated that weekly meat intake was positively correlated with depression symptoms. Darooghegi Mofrad et al. (20) have also reported that red meat consumption was associated with higher odds of depression, anxiety, and psychological distress in Tehrani women. In contrast, in Australian women, a “traditional” dietary pattern (characterized by meat and fish, along with vegetables, fruit, and whole grains) was associated with lower odds of depression and anxiety (14). Another cross-sectional study among 11,473 Chinese participants revealed consumption of meat (including fresh and salted meat and fish) less than once a week was significantly associated with increased risk of depression, compared with once a week or more meat intake (21). This effect might be due to family low income, insufficient consumption of fish, or perhaps anemia caused by low meat intake (39). A prospective cohort study with multistage random sampling has also found no significant relation between meat or processed meat intake and depressive symptoms in elders (28). A recently published systematic review has also demonstrated that the prevalence or risk of depression and/or anxiety was significantly greater in meat-abstainers compared to meat-consumers (19). These inconsistencies could be due to different study designs, different populations, and different tools and scales to measure variables.

Previous studies revealed that women were more susceptible to mental disorders; however, we found that red meat intake is related to depression symptoms in men, but not in women. This finding might be because of estrogens that have neuronal protective effects (40). In addition, our analyses showed significantly higher red meat intake in males than females (84.7 vs. 74.4 g/day, *p* < 0.001) that could be the reason.

Although, previous studies have shown that the prevalence of depression in overweight or obese individuals was higher than in normal-weight participants (41), our findings showed a positive relation between red meat intake and depression symptoms in normal-weight individuals and showed no relation in the overweight or obese participants. Furthermore, in overweight or obese participants, white meat intake showed an inverse association with psychological distress symptoms, whereas, there was no linkage in normal-weight individuals. Future prospective investigations are suggested to shed light on these different associations among overweight or obese individuals vs. normal-weight individuals in relation to depression. In the case of white meat intake, the current study was conducted in one of the central provinces of Iran that is far from the sea and fish was less likely to be consumed by the study population than chicken and poultry; this point might affect our findings. Hence, we made adjustment for omega-3 intake in the analysis. However, the results were still unexplained and further investigations are needed.

The evidence that explains mechanisms of the relation of meat intake and mental health is not completely understood, but some previous studies showed low-grade inflammatory status in depression and anxiety disorders (42–45). Inflammation plays a potential role in the etiology of depression (46); on the other hand, it has indicated that red meat intake could elevate levels of pro-inflammatory cytokines, such as C-reactive protein (CRP), tumor-necrosis factor alpha (TNF-α), and interleukin (IL)-6 (47). Unfortunately, in the current study, it was not possible to evaluate inflammatory and oxidative biomarkers. Although, the involved biochemical pathways are not fully known, some studies blamed total fat and fat types of meat for this condition (17, 48). Diets rich in saturated fatty acid (SFA) or total fats could increase free radical production and elevate oxidative stress and inflammation (49–51). Pro-inflammatory cytokines may disrupt neurotransmitter metabolism pathways, reduce plasma tryptophan level, and prevent brain-derived neurotrophic factor (BDNF) expression (52, 53). BNFD is a peptide critical for optimal neuronal function, which has been indicated to be decreased in depression (54, 55). In addition, endothelium is involved in synthesis and secretion of BDNF; therefore, endothelial dysfunction leading to disturb cell signaling cascades (56). It was suggested that diets high in meat, fish, and poultry increase the risk of inflammation and depression due to *n*-6 to *n*-3 fatty acid ratio (57). Plasma concentrations of tryptophan, an essential amino acid to produce serotonin, and large neutral amino acids (LNAAs) could be another mechanism, which may be involved in depression (58). Brain concentrations of tryptophan depend on plasma concentrations of both tryptophan and LNAA, which competes with tryptophan to cross over the blood–brain barrier (59). Some studies have shown a higher reduction in plasma tryptophan than plasma LNAA after consumption of a meal rich in proteins or proteins plus fats, such as meat (60).

The strengths of the present study were collecting data from a large population of Iranian adults and using validated questionnaires to evaluate psychological disorders, physical activity, and dietary intake. Effects of several potential confounders were also taken into account. However, several limitations need to be considered when interpreting our findings. Due to the cross-sectional design of the study, we did not have possibility to have causal relation between meat intake and mental disorders. Large prospective cohort studies are necessary to distinguish a causal relation. In addition, all data derived from FFQs might be invalid and physiologically implausible because memory and recall are not valid tools to collect scientific data (61). Since the number of lean participants was low in our sample (*n* = 114, 3.4%), we had to include these individuals in the category of normal-weight participants. The prevalence of depression and anxiety symptoms was higher in lean participants than normal-weight individuals (41.2 vs. 30% for depression symptoms and 16.7 vs. 15.0% for anxiety symptoms). Some sort of bias might occur due to this inclusion; however, it would be negligible, and such error would shift the ORs toward the null. Different types of red and white meat were not specifically investigated. Although, we were interested in examining the separate associations between processed meat and red meat intake and outcomes of interest in the current study, low consumption of processed red meat in the study population (6 ± 1 g/day) led us to consider total red meat, rather than processed meat and red meat intake, as the exposure. However, when we excluded processed meat and considered only unprocessed red meat intake, our results did not change. We could not measure biomarkers of inflammation and oxidative status. In addition, it was not possible to assess body resources of micronutrient, such as vitamin D, B_6_, B_12_, folate, and zinc, which might involve in mental disorders (18, 62–64). In addition, the study population consisted of a medical university non-academic staff, including crews, employees, and managers. Although the socio-economic status of the study population was representative of general Iranian population, generalization the findings to other populations should be made cautiously.

## Conclusions

We found that higher intake of red meat was associated with increased risk of depression symptoms, especially in males. In overweight or obese participants, white meat intake was inversely associated with psychological distress symptoms. Red meat intake was related to increased odds of depression symptoms in normal-weight participants. Further, prospective studies are needed to confirm these findings.

## Data Availability Statement

The raw data supporting the conclusions of this article will be made available by the authors, without undue reservation.

## Ethics Statement

The studies involving human participants were reviewed and approved by Isfahan University of Medical Sciences. The patients/participants provided their written informed consent to participate in this study.

## Author Contributions

SK, AK, PS, HA, AE, and PA contributed in conception, design, data collection, data interpretation, manuscript drafting, approval of the final version of the manuscript, and agreed for all aspects of the work. All authors contributed to the article and approved the submitted version.

## Conflict of Interest

The authors declare that the research was conducted in the absence of any commercial or financial relationships that could be construed as a potential conflict of interest.

## Publisher's Note

All claims expressed in this article are solely those of the authors and do not necessarily represent those of their affiliated organizations, or those of the publisher, the editors and the reviewers. Any product that may be evaluated in this article, or claim that may be made by its manufacturer, is not guaranteed or endorsed by the publisher.

## Abbreviations

ANCOVA: analysis of covariance
BMI: body mass index
BDNF: drain-derived neurotrophic factor
CES-D: Center for Epidemiologic Studies-Depression
FFQ: food frequency questionnaire
FGIDs: functional gastrointestinal disorders
GHQ: General Health Questionnaire
GPPAQ: General Practice Physical Activity Questionnaire
HADS: Hospital Anxiety and Depression Scale
IUMS: Isfahan University of Medical Sciences
LNAA: large neutral amino acids
OR: odds ratio
SEPAHAN: Study on the Epidemiology of Psychological-Alimentary Health and Nutrition
SES: socioeconomic status
SPSS: Statistical Package for Social Sciences.
