# Supplementary material for: Red and White Meat Intake in Relation to Mental Disorders in Iranian Adults
Source: Front Nutr. 2021 Jul 27;8:710555. doi: 10.3389/fnut.2021.710555 (PMC8353089; doi:10.3389/fnut.2021.710555)
Supplement: Supplementary file 1 [file Data_Sheet_1.docx]

**Online Supporting Materials**

| **Supplemental Table 1.** Multivariable-adjusted odds ratios and 95% confidence intervals for depression, anxiety and psychological distress symptoms across quartiles of energy-adjusted red and white meat intake in men^1^ | | | | | | | | | | |
| --- | --- | --- | --- | --- | --- | --- | --- | --- | --- | --- |
|  | Quartiles of red meat intake | | | | | Quartiles of white meat intake | | | | |
|  | Q1  (n=349) | Q2  (n=319) | Q3  (n=332) | Q4  (n=403) | p _trend_ | Q1  (n=381) | Q2  (n=309) | Q3  (n=300) | Q4  (n=413) | p _trend_ |
| Depression symptoms | |  |  |  |  |  |  |  |  |  |
| Crude | 1.00 | 1.15 (0.77-1.72) | 1.08 (0.73-1.60) | 1.75 (1.23-2.51) | <0.001 | 1.00 | 0.71 (0.48-1.05) | 1.16 (0.80-1.66) | 0.93 (0.66-1.31) | 0.77 |
| Model 1 | 1.00 | 1.37 (0.85-2.19) | 1.15 (0.71-1.84) | 1.82 (1.20-2.77) | 0.01 | 1.00 | 0.84 (0.52-1.34) | 1.53 (0.99-2.38) | 0.97 (0.65-1.46) | 0.58 |
| Model 2 | 1.00 | 1.43 (0.86-2.36) | 1.12 (0.66-1.88) | 1.92 (1.17-3.15) | 0.03 | 1.00 | 0.88 (0.54-1.46) | 1.64 (1.02-2.63) | 1.05 (0.65-1.68) | 0.40 |
| Anxiety symptoms |  |  |  |  |  |  |  |  |  |  |
| Crude | 1.00 | 1.30 (0.76-2.24) | 0.76 (0.42-1.39) | 1.28 (0.76-2.14) | 0.68 | 1.00 | 0.76 (0.43-1.34) | 1.13 (0.67-1.91) | 0.95 (0.58-1.57) | 0.86 |
| Model 1 | 1.00 | 1.51 (0.79-2.89) | 0.59 (0.28-1.24) | 1.10 (0.59-2.03) | 0.64 | 1.00 | 0.83 (0.41-1.66) | 1.25 (0.66-2.38) | 0.86 (0.47-1.57) | 0.86 |
| Model 2 | 1.00 | 1.80 (0.89-3.63) | 0.89 (0.39-2.02) | 1.62 (0.75-3.48) | 0.51 | 1.00 | 1.05 (0.50-2.19) | 1.43 (0.71-2.90) | 1.06 (0.52-2.15) | 0.70 |
| Psychological distress symptoms | | |  |  |  |  |  |  |  | |
| Crude | 1.00 | 0.89 (0.59-1.35) | 1.02 (0.68-1.52) | 1.00 (0.68-1.46) | 0.85 | 1.00 | 0.99 (0.66-1.49) | 1.28 (0.87-1.91) | 0.96 (0.66-1.41) | 0.89 |
| Model 1 | 1.00 | 1.03 (0.63-1.68) | 1.06 (0.66-1.71) | 1.08 (0.69-1.68) | 0.72 | 1.00 | 1.11 (0.68-1.82) | 1.65 (1.02-2.65) | 1.02 (0.65-1.59) | 0.62 |
| Model 2 | 1.00 | 1.14 (0.68-1.94) | 1.13 (0.66-1.93) | 1.18 (0.69-2.02) | 0.58 | 1.00 | 1.16 (0.67-2.00) | 2.02 (1.20-3.39) | 1.33 (0.79-2.24) | 0.11 |

^1^Model 1: Adjusted for age, energy intake, physical activity, smoking, marital status, education, socioeconomic status (SES), diabetes, intake of Anti-psychotic medications and dietary supplements.

Model 2: Additional controlling for dietary intake of high fat dairy, nuts, soy and legumes, grains, fruit and vegetables, n-3 fatty acids and BMI.

| **Supplemental Table 2.** Multivariable-adjusted odds ratios and 95% confidence intervals for depression, anxiety and psychological distress symptoms across quartiles of energy-adjusted red and white meat intake in women^1^ | | | | | | | | | | |
| --- | --- | --- | --- | --- | --- | --- | --- | --- | --- | --- |
|  | Quartiles of red meat intake | | | | | Quartiles white meat intake | | | | |
|  | Q1  (n=491) | Q2  (n=522) | Q3  (n=509) | Q4  (n=437) | p _trend_ | Q1  (n=495) | Q2  (n=532) | Q3  (n=541) | Q4  (n=427) | p _trend_ |
| Depression symptoms |  |  |  |  |  |  |  |  |  |  |
| Crude | 1.00 | 1.13 (0.87-1.47) | 1.21 (0.92-1.57) | 1.36 (1.04-1.79) | 0.02 | 1.00 | 0.96 (0.74-1.25) | 0.96 (0.74-1.25) | 0.95 (0.72-1.25) | 0.72 |
| Model 1 | 1.00 | 1.12 (0.83-1.51) | 1.21 (0.90-1.62) | 1.38 (1.02-1.86) | 0.04 | 1.00 | 1.01 (0.75-1.37) | 1.06 (0.78-1.43) | 0.89 (0.65-1.23) | 0.58 |
| Model 2 | 1.00 | 1.05 (0.77-1.44) | 1.16 (0.84-1.59) | 1.31 (0.93-1.84) | 0.11 | 1.00 | 0.96 (0.70-1.31) | 1.01 (0.73-1.39) | 0.82 (0.58-1.15) | 0.32 |
| Anxiety symptoms |  |  |  |  |  |  |  |  |  |  |
| Crude | 1.00 | 1.11 (0.79-1.55) | 0.97 (0.69-1.37) | 1.31 (0.93-1.84) | 0.23 | 1.00 | 1.15 (0.82-1.60) | 1.04 (0.74-1.45) | 0.97 (0.68-1.39) | 0.73 |
| Model 1 | 1.00 | 0.95 (0.65-1.38) | 0.85 (0.58-1.25) | 1.19 (0.82-1.73) | 0.50 | 1.00 | 1.16 (0.80-1.70) | 1.17 (0.79-1.71) | 0.91 (0.61-1.38) | 0.69 |
| Model 2 | 1.00 | 0.92 (0.62-1.37) | 0.89 (0.59-1.34) | 1.25 (0.82-1.91) | 0.35 | 1.00 | 1.15 (0.78-1.70) | 1.17 (0.78-1.75) | 0.92 (0.59-1.44) | 0.77 |
| Psychological distress symptoms | | |  |  |  |  |  |  |  | |
| Crude | 1.00 | 1.03 (0.78-1.35) | 0.93 (0.70-1.24) | 1.05 (0.79-1.41) | 0.92 | 1.00 | 0.88 (0.67-1.16) | 0.84 (0.63-1.11) | 0.78 (0.58-1.06) | 0.10 |
| Model 1 | 1.00 | 1.01 (0.74-1.37) | 0.97 (0.71-1.32) | 1.02 (0.74-1.40) | 0.99 | 1.00 | 0.91 (0.66-1.24) | 0.94 (0.69-1.30) | 0.80 (0.57-1.11) | 0.24 |
| Model 2 | 1.00 | 0.95 (0.68-1.31) | 0.99 (0.71-1.38) | 1.02 (0.71-1.46) | 0.86 | 1.00 | 0.86 (0.62-1.19) | 0.92 (0.66-1.28) | 0.73 (0.51-1.04) | 0.14 |

^1^Model 1: Adjusted for age, energy intake, physical activity, smoking, marital status, education, socioeconomic status (SES), diabetes, intake of Anti-psychotic medications and dietary supplements.

Model 2: Additional controlling for dietary intake of high fat dairy, nuts, soy and legumes, grains, fruit and vegetables, n-3 fatty acids and BMI.

| **Supplemental Table 3.** Multivariable-adjusted odds ratios and 95% confidence intervals for depression, anxiety and psychological distress symptoms across quartiles of energy-adjusted red and white meat intake in overweight or obese participants (BMI≥25kg/m^2^)^1^ | | | | | | | | | | |
| --- | --- | --- | --- | --- | --- | --- | --- | --- | --- | --- |
|  | Quartiles of red meat intake | | | | | Quartiles of white meat intake | | | | |
|  | Q1  (n=367) | Q2  (n=372) | Q3  (n=383) | Q4  (n=384) | p _trend_ | Q1  (n=384) | Q2  (n=374) | Q3  (n=377) | Q4  (n=371) | p _trend_ |
| Depression symptoms | |  |  |  |  |  |  |  |  |  |
| Crude | 1.00 | 1.07 (0.77-1.50) | 1.29 (0.93-1.79) | 1.43 (1.04-1.97) | 0.02 | 1.00 | 0.92 (0.67-1.26) | 0.95 (0.69-1.30) | 0.70 (0.56-1.07) | 0.16 |
| Model 1 | 1.00 | 1.01 (0.69-1.47) | 1.19 (0.82-1.72) | 1.41 (0.98-2.02) | 0.04 | 1.00 | 0.98 (0.68-1.41) | 1.13 (0.78-1.63) | 0.73 (0.51-1.06) | 0.20 |
| Model 2 | 1.00 | 0.98 (0.67-1.44) | 1.13 (0.77-1.65) | 1.27 (0.86-1.90) | 0.18 | 1.00 | 0.97 (0.67-1.40) | 1.08 (0.74-1.57) | 0.68 (0.45-1.00) | 0.11 |
| Anxiety symptoms | |  |  |  |  |  |  |  |  |  |
| Crude | 1.00 | 1.18 (0.77-1.80) | 0.98 (0.64-1.52) | 1.39 (0.92-2.09) | 0.21 | 1.00 | 0.92 (0.61-1.38) | 1.06 (0.71-1.58) | 0.75 (0.49-1.14) | 0.31 |
| Model 1 | 1.00 | 0.88 (0.54-1.44) | 0.76 (0.46-1.26) | 1.31 (0.82-2.09) | 0.33 | 1.00 | 0.93 (0.58-1.49) | 1.14 (0.71-1.83) | 0.67 (0.40-1.11) | 0.26 |
| Model 2 | 1.00 | 0.96 (0.58-1.58) | 0.86 (0.51-1.45) | 1.61 (0.96-2.71) | 0.11 | 1.00 | 0.96 (0.59-1.57) | 1.21 (0.74-1.97) | 0.73 (0.42-1.24) | 0.47 |
| Psychological distress symptoms | | |  |  |  |  |  |  |  |  |
| Crude | 1.00 | 0.73 (0.51-1.03) | 0.87 (0.62-1.21) | 0.85 (0.60-1.19) | 0.55 | 1.00 | 0.90 (0.64-1.25) | 0.85 (0.60-1.19) | 0.66 (0.46-0.94) | 0.02 |
| Model 1 | 1.00 | 0.77 (0.52-1.14) | 0.91 (0.62-1.34) | 0.88 (0.60-1.29) | 0.70 | 1.00 | 0.95 (0.64-1.40) | 1.09 (0.74-1.62) | 0.68 (0.46-1.02) | 0.14 |
| Model 2 | 1.00 | 0.78 (0.52-1.16) | 0.90 (0.60-1.34) | 0.83 (0.54-1.27) | 0.52 | 1.00 | 0.96 (0.65-1.43) | 1.06 (0.71-1.59) | 0.64 (0.42-0.99) | 0.09 |

^1^Model 1: Adjusted for age, sex, energy intake, physical activity, smoking, marital status, education, socioeconomic status (SES), diabetes, intake of Anti-psychotic medications and dietary supplements.

Model 2: Additional controlling for dietary intake of high fat dairy, nuts, soy and legumes, grains, fruit and vegetables and n-3 fatty acids.

| **Supplemental Table 4.** Multivariable-adjusted odds ratios and 95% confidence intervals for depression, anxiety and psychological distress symptoms across quartiles of energy-adjusted red and white meat intake in normal-weight participants (BMI<25kg/m^2^)^1^ | | | | | | | | | | |
| --- | --- | --- | --- | --- | --- | --- | --- | --- | --- | --- |
|  | Quartiles of red meat intake | | | | | Quartiles of white meat intake | | | | |
|  | Q1  (n=473) | Q2  (n=469) | Q3  (n=458) | Q4  (n=456) | p _trend_ | Q1  (n=456) | Q2  (n=467) | Q3  (n=464) | Q4  (n=469) | p _trend_ |
| Depression symptoms | |  |  |  |  |  |  |  |  |  |
| Crude | 1.00 | 1.24 (0.93-1.66) | 1.08 (0.81-1.46) | 1.44 (1.08-1.92) | 0.04 | 1.00 | 0.94 (0.70-1.26) | 1.20 (0.90-1.60) | 1.06 (0.79-1.41) | 0.37 |
| Model 1 | 1.00 | 1.33 (0.95-1.87) | 1.18 (0.84-1.66) | 1.55 (1.11-2.16) | 0.03 | 1.00 | 0.97 (0.69-1.36) | 1.23 (0.87-1.73) | 1.11 (0.79-1.55) | 0.30 |
| Model 2 | 1.00 | 1.40 (0.99-2.00) | 1.24 (0.87-1.79) | 1.66 (1.14-2.42) | 0.03 | 1.00 | 0.99 (0.70-1.41) | 1.29 (0.91-1.85) | 1.20 (0.83-1.74) | 0.15 |
| Anxiety symptoms | |  |  |  |  |  |  |  |  |  |
| Crude | 1.00 | 1.20 (0.82-1.75) | 0.89 (0.60-1.33) | 1.10 (0.75-1.62) | 0.99 | 1.00 | 1.31 (0.88-1.93) | 1.21 (0.81-1.80) | 1.15 (0.77-1.72) | 0.62 |
| Model 1 | 1.00 | 1.22 (0.79-1.89) | 0.83 (0.52-1.30) | 0.96 (0.62-1.50) | 0.46 | 1.00 | 1.23 (0.78-1.94) | 1.24 (0.78-1.97) | 1.14 (0.72-1.81) | 0.60 |
| Model 2 | 1.00 | 1.24 (0.79-1.96) | 0.84 (0.51-1.36) | 0.95 (0.57-1.56) | 0.44 | 1.00 | 1.32 (0.82-2.11) | 1.36 (0.84-2.20) | 1.31 (0.79-2.17) | 0.32 |
| Psychological distress symptoms | | |  |  |  |  |  |  | |  |
| Crude | 1.00 | 1.28 (0.95-1.73) | 1.07 (0.78-1.46) | 1.13 (0.83-1.54) | 0.72 | 1.00 | 1.04 (0.77-1.42) | 1.18 (0.87-1.60) | 0.99 (0.73-1.35) | 0.85 |
| Model 1 | 1.00 | 1.28 (0.90-1.82) | 1.07 (0.75-1.53) | 1.17 (0.82-1.65) | 0.63 | 1.00 | 0.97 (0.68-1.38) | 1.13 (0.79-1.62) | 1.04 (0.73-1.48) | 0.63 |
| Model 2 | 1.00 | 1.32 (0.92-1.89) | 1.10 (0.76-1.60) | 1.18 (0.80-1.75) | 0.66 | 1.00 | 0.98 (0.68-1.42) | 1.18 (0.81-1.70) | 1.10 (0.75-1.61) | 0.44 |

^1^Model 1: Adjusted for age, sex, energy intake, physical activity, smoking, marital status, education, socioeconomic status (SES), diabetes, intake of Anti-psychotic medications and dietary supplements.

Model 2: Additional controlling for dietary intake of high fat dairy, nuts, soy and legumes, grains, fruit and vegetables and n-3 fatty acids
